# Biogenic Fabrication of Silver Nanoparticles Using *Calotropis procera* Flower Extract with Enhanced Biomimetics Attributes

**DOI:** 10.3390/ma16114058

**Published:** 2023-05-30

**Authors:** Pooja V. Nagime, Sudarshan Singh, Nishat M. Shaikh, Komal S. Gomare, Havagiray Chitme, Basel A. Abdel-Wahab, Yahya S. Alqahtany, Masood Medleri Khateeb, Mohammed Shafiuddin Habeeb, Marwa B. Bakir

**Affiliations:** 1 Department of Biotechnology, Dayanand Science College, Latur 413512, India; nishatshaikh2017@gmail.com (N.M.S.); komalgomare2007@gmail.com (K.S.G.); 2 Department of Pharmaceutical Sciences, Faculty of Pharmacy, Chiang Mai University, Chiang Mai 50200, Thailand; sudarshansingh83@hotmail.com; 3 Faculty of Pharmacy, DIT University, Dehradun 248009, India; 4 Department of Pharmacology, College of Pharmacy, Najran University, Najran P.O. Box 1988, Saudi Arabia; babdelnaem@nu.edu.sa (B.A.A.-W.); mmkhateeb@nu.edu.sa (M.M.K.); mshabeeb@nu.edu.sa (M.S.H.); 5 Department of Pharmaceutical Chemistry, College of Pharmacy, Najran University, Najran P.O. Box 1988, Saudi Arabia; ysalqahtany@nu.edu.sa; 6 Department of Pharmacology, College of Medicine, Najran University, Najran P.O. Box 1988, Saudi Arabia; mbbakir@nu.edu.sa

**Keywords:** antibacterial, antioxidant, anti-inflammatory, antidiabetic, *Calotropis procera*, silver nanoparticles

## Abstract

There have been some reports demonstrating the biogenic synthesis of silver nanoparticles (AgNPs) using *Calotropis procera* (CP) plant extract; however, detailed in-depth debriefing of the vital synthesis parameter for rapid, facile, efficacious synthesis at varied temperatures with effectual characterization of nanoparticles and biomimetic attribute is lacking. This study presents a comprehensive demarcation of the sustainable fabrication of biogenic *C. procera* flower extract capped and stabilized silver nanoparticles (CP-AgNPs) synthesis with thorough phytochemical characterization and potential biological application. The results revealed that the successful synthesis of CP-AgNPs was instantaneous with the maximum intensity of the plasmonic peak ~400 nm, while morphological results revealed the cubic shape of nanoparticles. CP-AgNPs were found to present stable, well-dispersed, uniform, high anionic zeta potential, and crystalline nanoparticles with a crystallite size of ~23.8 nm. The FTIR spectra indicated that CP-AgNPs were properly capped by the bioactive of *C. procera*. Moreover, the synthesized CP-AgNPs exhibited hydrogen peroxide scavenging efficacy. In addition, CP-AgNPs showed antibacterial and antifungal activity against pathogenic bacteria. CP-AgNPs displayed significant in vitro antidiabetic and anti-inflammatory activity. An efficient and convenient approach for synthesizing AgNPs using *C. procera* flower has been developed with enhanced biomimetic attributes that may be further utilized for water treatment, biosensors, biomedicine, and in allied science.

## 1. Introduction

Nanoparticles (NPs) are tiny materials with a size range of 1–100 nm [1]. The modification in size and shape of NPs depends on various properties such as temperature, the redox potential of synthesized particles, color, the conductivity of the material, chemical stability, electrical qualities, optics qualities, and electrical properties [2]. In the fields of medicine [3], cosmetics [4], sensing [5,6], the food industry [7,8,9], surgical devices [10,11], and the chemical industry [12], the size and shape of materials have distinct uses. Metallic NPs (MNPs) are synthesized using a variety of processes, including physical, chemical, and biological approaches [13]. Laser ablation [14], inert gas condensation [15], electric arc discharge [16], and radiofrequency plasma technique [17] are the basic physical processes used to fabricate AgNPs. However, the physical approach to the synthesis MNPs has several drawbacks, including energy requirement, necessitating a lengthy process to produce thermal energy, consuming a substantial quantity of energy by raising the temperature of the environment and occupying a considerable surface area that makes this approach of synthesizing MNPs ineffective. While chemically synthesized MNPs require toxic reducing agents such as sodium citrate, boron hydrate, and organic solvents, which are highly detrimental to the environment [18], the most advantageous way for synthesizing MNPs is biological rather than physical or chemical. Biosynthesis of MNPs employing microorganisms such as algae, fungi, bacteria, and plants have been reported in several investigations [12]. The primary disadvantage of employing microorganisms to synthesize MNPs is their complexity, potential for contamination, tedious process, and difficulty in maintaining large-scale colonies [19]. There are two techniques reported in synthesizing MNPs including the top-down approach and the bottom-up approach generally used in the biological synthesis of MNPs. The breakdown of bulk material to nanoscale using various lithographic techniques, such as grinding, mechanical milling, chemical etching, sputtering, and pulsed laser deposition, is known as a top-down approach. MNPs are commonly synthesized biologically via the bottom-up approach that utilizes self-assembling atoms to produce nuclei, which are then transformed into MNPs.

Several studies indicated that phenolic and flavonoid-rich plant extract, including a leaf, root, fruit, flower, etc., can be used in the green synthesis of MNPs. Biogenic synthesis of MNPs has several advantages of being environmentally sustainable, less expensive, less time-consuming, causing no harm to the environment, accessible precursors, and having a great production capacity. Green synthesis of various MNPs, including zinc oxide, gold, silver, palladium, iron, and copper, is well documented [20]. Alkaloids, flavonoids, glycosides, saponins, tannins, terpenoids, phenol, carbohydrates, and proteins are just a few of the phytochemicals that are found in plants responsible for the formation of MNPs [12]. Among the several types of MNPs, AgNPs have drawn the most attention due to their unique physical, chemical, and biological characteristics, as well as their several therapeutic benefits, including antimicrobial, anti-cancer, and anti-inflammatory activities [1].

*Calotropis procera* is an evergreen xerophytic plant (Apocynaceae) typically found in dry and semi-dry areas [21]. In the past, the pharmacological properties of *C. procera* were widely used to treat human illnesses including dysentery, indigestion, diarrhea, elephantiasis, indigestion, fever, leprosy, asthma, rheumatism, and dermatitis [21]. Numerous studies have revealed the presence of metabolites in diverse plant sections, including alkaloids, flavonoids, steroids, tannins, terpenoids, saponins, and cardiac glycosides, which make suitable materials for the green synthesis of metal ions [12]. Therefore, the research centered on the biogenic synthesis of AgNPs employing the aqueous flowers extract of the *C. procera*, as well as the investigation of the synthesized AgNPs that were characterized and optimized by employing various approaches for the assessment of anti-bacterial, anti-fungal, antioxidant, anti-inflammatory, and anti-diabetic activities.

## 2. Materials and Methods

All reagents used for the experiment were analytical grade purchased from Himedia (HiMedia Laboratories Pvt. Ltd., Mumbai, India) including silver nitrate (Mw: 169.87 g/mol; and purity: ≥99.9%), hydrogen peroxide, iodine, potassium iodide (Mw 166.00 g/mol and purity: 99%), sodium hydroxide (M: 39.997 g/mol and purity: 97%), sulfuric acid, chloroform, benzene, ammonia, naphthol, ethanol, copper acetate, ferric chloride (Mw: 162.2 g/mol and purity: ≥99%), Butylated hydroxytoluene (BHT), hydrochloric acid, potato dextrose agar, nutrient agar, potassium ferricyanide, TCA, α-amylase, etc. Bacterial culture including *Bacillus subtilis* (MTCC 121T), *Shigella flexneri* (MTCC 1457), *Bacillus megaterium* (NCTC 6094), *Trichoderma viride*, *Aspergillus niger* (MTCC 12975), and *Penicillium crysogenum* (NCPF 2802) were collected from Dayanand Science College, Latur, India.

### 2.1. Collection of Plant Material, Extract Preparation, and Phytochemical Analysis

*Calotropis procera* were collected from the local city area (Latur, Maharashtra, India) and then identified from the botany department of Dayanand Science College, Latur, India (DSC-HB-CP-flower-54-2023). Fresh *C. procera* flowers were washed and dried at room temperature for 10 to 15 days. Briefly, 450 mL of water were added to 50 g of flower powder in a 500 mL conical flask. To avoid the solution’s reaction to light, it was covered with aluminum foil. The mixture was then shaken for 30 min using a mechanical shaker, heated on a hotplate at 65 °C for 1 h with a magnetic stirrer, and then allowed to cool at room temperature, referred to as *C. procera* flower extract (CP). The extract was further tested for the presence of phytochemicals such as alkaloids, flavonoids, carbohydrates, glycosides, phlobatanin, anthocyanin, anthroquinones, diterpenes, proteins, saponins, tannins, and terpenoids [3].

### 2.2. Synthesis and Characterization of AgNPs

Biogenic AgNPs were synthesized using CP as a reductant, capping, and stabilizer. In brief, the ideal volume (200 mL) of aqueous flower extract was mixed with 100 mL of silver nitrate solution (5 mM) to biosynthesize AgNPs and stirred in the dark for 4 h at 40 °C to prevent auto-oxidation. The synthesized AgNPs were centrifuged for 15 min at 15,000 rpm using a Remi centrifuge (CM-12 plus Wasai, Maharashtra, India) to obtain reduced pellets of CP-AgNPs and redispersed in distilled water. Moreover, the synthesized CP-AgNPs were studied concerning concentration, reaction time, temperature, and volume ratio. The AgNPs were synthesized using several experimental variables, including reaction time (0 h, 2 h, 4 h, 8 h, and 16 h), temperature (4 °C, 20 °C, 40 °C, 60 °C, and 80 °C), silver ion solution concentration (1 mM, 2.5 mM, 5 mM, 7.5 mM, and 10 mM), and the volume ratio of silver ion solution to *C. provera* flower extract (1:1, 1:2, 1:3, 1:4, and 1:5) to further optimize the condition and investigate the effects of variables. UV-Vis spectrophotometer data were used to keep a record of these experimental variables. Furthermore, AgNPs were vacuum-dried at 60 °C for auxiliary characterization and stored at room temperature in a vacuum till further use.

The effect of surface plasmon resonance (SPR) was examined throughout the biosynthesis of AgNPs and the reduction of silver ions using a double beam UV-Vis spectrophotometer (Agilent Technologies, Cary 60 UV-Vis; Santa Clara, CA, USA) in the wavelength range of 280–780 nm monitored. The organic functional groups attached to the surface of the silver nanoparticles that operate as the capping, stabilizing, and reducing agents were identified using the FTIR spectrometer (Perkin Elmer (L1600401), Middlewich, Cheshire, UK) and recorded in the 4000–400 cm^−1^ range. Further, the morphology and size of CP-AgNPs were studied using Transmission electron microscopy (TEM-JEOL, USA) at 50,000× magnification. Dynamic light scattering (DLS) and zeta potential measurements were carried out for CP-AgNPs using a Malvern DLS instrument zetasizer. Furthermore, the field-emission scanning electron microscope (FE-SEM) with ultra-high-resolution at low-voltage imaging with unique low-vacuum capabilities (FEI Nova NanoSEM 450, Suite 1001 New York, NY, USA) was used at a 10 kV accelerating voltage and 100,000× magnifications to capture the surface morphology of AgNPs. In addition, the FE-SEM images and size distribution of the generated CP-AgNPs were examined using xT microscope control v6.3.2 equipped with 3189 software. The crystalline character of the biosynthesized AgNPs was further revealed through XRD analysis utilizing an advanced X-ray diffractometer (XRD-7000 X-ray diffractometer, Shimadzu Corporation, Chiyoda-ku, Tokyo, Japan) with Cu-K radiation of wavelength 1.5406 Ǻ and a scanning angle 2θ from 20° to 0°. Furthermore, the Debey–Scherrer equation was applied to determine the particle sizes.

D = 0.9λ/(βcosθ)

where D stands for the size of the crystal (nm), λ is the X-ray wavelength, θ represents Bragg’s angle in radians, and β is the whole width at half the maximum of the peak in radians.

### 2.3. Biological Activities of CP-AgNPs

#### 2.3.1. Antibacterial Activity

The Agar well diffusion method was used to study the antibacterial activity of the CP-AgNPs [22]. Bacteria including *Bacillus subtilis*, *Shigella flexneri*, and *Bacillus megaterium* were isolated using a nutrient agar medium. Subsequently, 28 g of nutrient agar powder were dissolved in 1000 mL of distilled water, and the prepared media was autoclaved (LSC-05 Labline Stock Centre Mumbai, Maharashtra, India) for 15 min at 121 °C. The medium was thereafter placed into petri plates and held there to solidify in an incubator (Remi elektro technik 372-LAG, Mumbai, Maharashtra, India). In each well, 100 µL from stock of CP-AgNPs were then transferred. Antibiotics penicillin was employed as the positive control. The nutrient agar plates were maintained for 30 min in the refrigerator (Godrej, Maharashtra, India) for the diffusion of AgNPs. For 24 h, the plates were incubated at 37 °C, and the zone of inhibition was measured using an antibiotic zone scale (Himedia, Maharashtra, India).

#### 2.3.2. Antifungal Activity

The antifungal activity of synthesized CP-AgNPs was investigated against *Trichoderma viride*, *Aspergillus niger*, and *Penicillium crysogenum* using a well diffusion method [23]. Briefly, approximately 39 g of powder potato dextrose agar were dissolved in 1000 mL of distilled water and autoclaved (LSC-05 Labline Stock Centre, Mumbai, Maharashtra, India) for 15 min at 121 °C. The media was thereafter placed in a sterile petri dish and left to solidify in an incubator (Remi elektro technik 372-LAG, Mumbai, Maharashtra, India). Fresh fungus culture was spread on the plates using cotton swabs. Wells were made using a borer (1.4 mm) and filled with 100 µL from stock of CP-AgNPs. Ampicillin and streptomycin were used as the positive control. For 30 min, the prepared agar plates were kept in the refrigerator (Godrej, Maharashtra, India) and later maintained in an incubator (Remi elektro technik 372-LAG, Maharashtra, India) for 24 h at 37 °C. The inhibitory zone around the well was measured using an antibiotic zone scale (Himedia, Mumbai, Maharashtra, India).

#### 2.3.3. Antioxidant Activity of CP-AgNPs

##### Hydrogen Peroxide Scavenging Activity

Hydrogen peroxide (H_2_O_2_) scavenging activity was accomplished by mixing 50 µL of 5 mM H_2_O_2_ solution with 10 mL from stock of CP-AgNPs and ascorbic acid as the positive control. The mixture was then incubated for 20 min at room temperature, and the absorbance of the prepared solutions was measured at 610 nm using a spectrophotometer (Spectramax M3, Thermo Scientific, Waltham, MA, USA) [24]. The percentage of hydrogen peroxide scavenging activity was calculated using the equation below.

$$\mathrm{Hydrogen}\;\mathrm{peroxide}\;\mathrm{scavenging}\;\left(\%\right)=\frac{Ab_{control}-Ab_{test}}{Ab_{control}}\times 100$$

where Ab_control_ is absorbance control and Ab_test_ is absorbance with CP-AgNPS.

##### Reducing Power Activity of CP-AgNPs

The reducing power of CP-AgNPs was determined as reported [25]. Briefly, the mixture of 2.5 mL of 200 mM phosphate buffer (pH 6.6) and 2.5 mL of 1% potassium ferricyanide was added to 10 mL of a centrifuged stock solution of CP-AgNPs. After being incubated at 50 °C for 20 min, the mixture was cooled down. About 2.5 mL of 10% TCA were then added to the mixture and centrifuged for 8 min at 3000 rpm using Remi of centrifuge (CM-12 plus, Maharashtra, India). The supernatant was collected and mixed with an equal volume of distilled water followed by the addition of 0.1% ferric chloride. The solution’s absorbance at 700 nm was measured using a spectrophotometer (Spectramax M3, Thermo Scientific, Waltham, MA, USA), and the percentage-reducing power of CP-AgNPs was calculated using an equation.

$$\mathrm{Reducing}\;\mathrm{power}\;\left(\%\right)=\frac{Ab_{control}-Ab_{test}}{Ab_{control}}\times 100$$

where Ab_control_ is the absorbance of control and Ab_test_ is absorbance with CP-AgNPS.

#### 2.3.4. Antidiabetic Activity of CP-AgNPs

In vitro, antidiabetic activity of CP-AgNPs was determined as reported [6]. Concisely, 250 mL of 2.0% (*w*/*v*) starch and 250 mL of the solution containing 1 U/mL of α-amylase were transferred to 250 mL of the flask containing stock solution of CP-AgNPs. The mixture was vortexed and incubated for 3 min at 20 °C. After, incubation of 500 µL of dinitro-salicylic acid was added to terminate the enzyme reaction. The obtained mixture was kept over boiling water bath (LSC-70 Labline Stock Centre, Mumbai, Maharashtra, India) and admixed with 250 µL of 1U/mL α-amylase. The solution was heated for 15 min before cooling for 3 min. Subsequently, 4500 αL of distilled water were added to the obtained volume and make-upped to 6000 µL with homogeneous mixing. The α-amylase inhibition activity was estimated using a spectrophotometer (Spectramax M3, Thermo Scientific, Waltham, MA, USA) at 540 nm. The percentage of the inhibition was calculated using the equation below.

$$\alpha \;\mathrm{amylase}\;\mathrm{inhibition}\;\left(\%\right)=\frac{Ab_{control}-Ab_{test}}{Ab_{control}}\times 100$$

where Ab_control_ is absorbance of control (the control α-amylase at 1 U/mL without any inhibitor represented the 100% enzyme activity) and Ab_test_ is absorbance with CP-AgNPS and standard acarbose, respectively.

#### 2.3.5. Anti-Inflammatory Activity of AgNPs

##### Inhibition of Protein Denaturation

In vitro, protein denaturation was quantified as reported [26]. In brief, CP-AgNPs and 1% of bovine albumin solution were admixed and pH 7.4 was adjusted using HCl. After 20 min of incubation at 37 °C, the mixture was heated for another 20 min at 51 °C. The absorbance at 660 nm was measured using a spectrophotometer (Spectramax M3, Thermo Scientific, Waltham, MA, USA). The percentage of the protein denaturation was calculated using the equation below.

$$\mathrm{Protein}\;\mathrm{denaturation}\;\left(\%\right)=\frac{Ab_{control}-Ab_{test}}{Ab_{control}}\times 100$$

where Ab_control_ is absorbance without extract and Ab_test_ is absorbance with CP-AgNPS.

##### Membrane Stabilization Activity

Fresh blood from the pathological lab of the hospital (Nilangekar Hospital, Maharashtra, India) was collected and centrifuged at 3000 rpm using a centrifuge (CM-12 plus, Maharashtra, India) for 10 min and rinsed with a similar volume of saline. Blood was restored for the red blood cell (RBC) suspension at a 10% volume ratio with normal saline. In the reaction mixture, 1 mL each of 10% of RBC suspension and test sample was admixed, while in the control, saline was replaced with the test sample, and Aspirin was tested as a standard. The test and control samples were incubated for 30 min in the water bath at 56 °C. The samples were cooled down and centrifuged using centrifuge (CM-12 plus, Maharashtra, India) at 2500 rpm for 5 min followed by absorbance measurement at 560 nm using a spectrophotometer [4]. The percentage of membrane stabilization was calculated using the equation below.

$$\mathrm{Membrane}\;\mathrm{stabilization}\;\left(\%\right)=\frac{Ab_{control}-Ab_{test}}{Ab_{control}}\times 100$$

where Ab_control_ is absorbance with aspirin and Ab_test_ is absorbance with CP-AgNPS.

## 3. Results

### 3.1. Phytochemical Analysis of the C. procera Flower Extract

*Calotropis procera* is an important Ayurvedic plant with significant medicinal properties used to treat several diseases. The *C. procera* plant extract from different parts has been reported to have significant therapeutic values. Additionally, *C. procera* is also used as a homeopathic medicine [27]. The hot maceration approach yielded high content of light brown *C. procera* extract. The result of the phytochemical analysis is presented in Supplementary Table S1 and Figure S1. The phytochemical profiling indicated that the *C. procera* aqueous flower extract contains alkaloids, anthraquinones, diterpenes, glycosides, phenols, phlobatanin, proteins, and terpenoids. The reduction, capping, and stability of CP-AgNPs are greatly aided by the presence of bioactive chemicals. Previous investigations demonstrated the presence of terpenoids, flavonoids, saponins, steroids, and cardiac glycopyrasides in *C. procera* aqueous flower extract [28].

### 3.2. Synthesis CP-AgNPs

**Figure 1:**
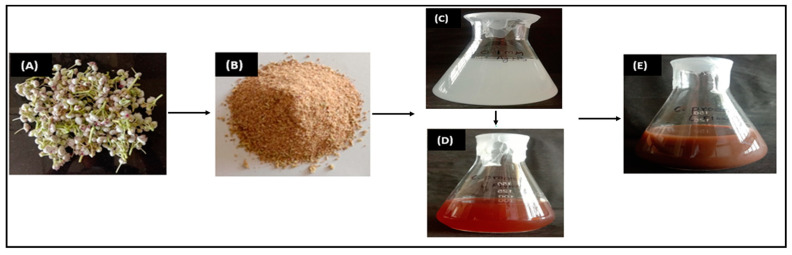
Systemic illustration of green synthesis of AgNPs using *C. provera* flower extract. The figure represents the process of fabrication as collection of fresh *C. provera* flower (**A**), followed with aqueous extraction and drying to powder form (**B**), further used for biogenic reduction of AgNO_3_ solution (**C**), to process fabrication of metallic nanoparticles using *C. provera* flower extract to AgNPs (**D**), in dark brown color as CP-AgNPs (**E**).

It is well known that the efficacy of phytoconstituent-mediated biogenic synthesis of MNPs is a function of the type and content of bioactive acting as a capping, reducing, and/or stabilizing agent. Phenolic-rich plants and extracts had previously been reported as effective capping or reducer/stabilizers of silver and gold nanoparticles [9]. In this study, phytoconstituents-rich *C. procera* aqueous flower extract was prepared in a facile and eco-friendly approach that did not require any toxic reagent. Silver nitrate and *C. procera* aqueous flower extract in a ratio of 1:2 successfully synthesized AgNPs (Figure 1), while AgNO_3_ serves as a precursor and extracts as a reducer or capping agent. The AgNO_3_ solution was initially colorless; however, the addition of *C. procera* aqueous flower extract turned the mixture into a dark brown color after incubation at room temperature. The bio-reduction of silver ions demonstrated a distinctive surface plasmon resonance ~400 nm in UV spectroscopy spectra. The presence of distinctive surface plasmon resonance (SPR) confirmed the synthesis of CP-reduced AgNPs [29]. Due to electron excitation and changes in electrical energy levels, the synthesized AgNPs became dark brown in an aqueous solution, suggesting the reduction of Ag+ into Ag° [1]. The effects of several parameters, including temperature, reaction time, silver nitrate concentration, and volume of *C. procera* aqueous flower extract, on the synthesis of AgNPs were investigated. AgNPs synthesis significantly relies on an operational parameter, regardless of the technology employed.

#### 3.2.1. Effect of Silver Nitrate Concentration on the Synthesis of AgNPs

**Figure 2:**
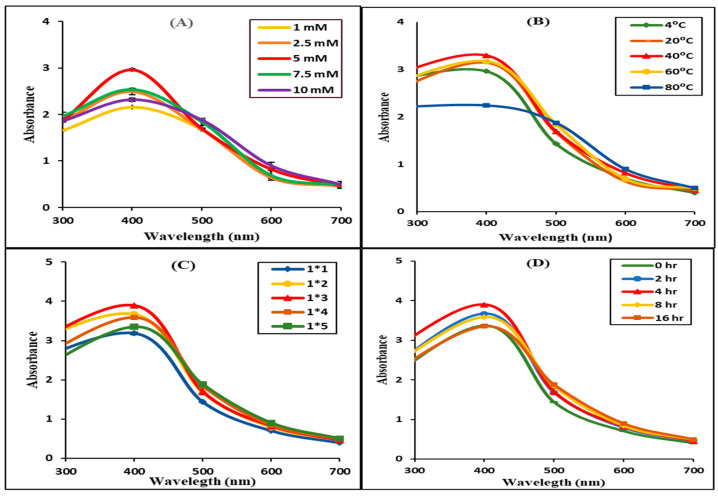
Biogenic synthesis of AgNPs using *C. procera* flower extract at various concentrations of AgNO_3_ (1 mM, 2.5 mM, 5 mM, 7.5 mM, and 10 mM) were studied, and the results were related to their UV-Vis absorption spectra (**A**). Furthermore, the effect of temperature (4 °C, 20 °C, 40 °C, 60 °C, and 80 °C) (**B**), varied ratio of 5 mM AgNO_3_ solution to *C. procera* flower extract (1*1, 1*2, 1*3, 1*4, and 1*5 *v*/*v*) (**C**), and reaction time (0 h, 4 h, 8 h, and 16 h) (**D**), on the fabrication of silver nanoparticles were studied for surface plasmon reference using UV-Visible spectroscopy.

The effect of AgNO_3_ concentration on the synthesis of AgNPs is depicted in Figure 2A. UV-Vis spectroscopy was used in this investigation to monitor the physiological reduction of silver ions to AgNPs. The reduction, production, and optimization of AgNO_3_ to AgNPs in aqueous solutions was accessed using UV-Vis spectroscopy in the scanning range of 300–700 nm. Several investigations indicated that the conversion of colorless AgNO_3_ to varied forms of brown color occurs due to the emergence of a distinctive surface plasmon resonance (SPR) peak of AgNPs at 400 nm. The results indicated development of a peak ~400 nm resulting due to reduction AgNO_3_ tested at 5 mM. Similar optimum observations were observed for AgNPs synthesized using *Erythrina abyssinica* aerial parts [30], while in a previous report plant leaf extract demonstrated that 5 mM and 4 mM were optimized concentrations, respectively [31]. The results showed that the concentration of metal ions strongly affects the formation of MNPs, avoiding the use of the excess amount of expensive precious metal.

#### 3.2.2. Effect of *Calotropis procera* Flower Extract Volume on the Synthesis of AgNPs

By altering the ratio of the extract (1:1, 2:1, 3:1, 4:1) in *v*/*v* with respect to fixed volume of AgNO_3_ solution (5 mM) in *v*/*v*, the AgNPs size and form were explored. The UV-Vis spectra indicated that at the ratio of 1:3 maximum absorption peak was measured at ~400 nm (Figure 2C). However, in another investigation, a 1:2 volume ratio of AgNO_3_, in a similar study using *Hagenia abyssinica*, demonstrated an ideal condition for the synthesis of AgNPs with an absorption peak of ~406 nm [31].

#### 3.2.3. Effect of Temperature on the Synthesis of CP-AgNPs

By employing a UV spectrometer, the effect of temperature on the development of CP-AgNPs was investigated. The results for the effect of temperature on biogenic synthesis of CP-AgNPs indicated that the color shifted from red to dark brown while the SPR was observed at highest absorption of ~400 nm at 40 °C (Figure 2B). Furthermore, based on the SPR absorption spectra and the quantity of AgNPs synthesized, 40 °C has been opted for as the optimal condition for the synthesis of CP-AgNPs. Similarly, the optimal temperature for the synthesis of AgNPs using Tridax Procumbent leaf extract was 40 °C reported in a previous study [32]. However, the optimal temperature for the synthesis of AgNPs from *Erythrina abyssinica* aerial parts of 80 °C has been reported [30]. At high temperatures, the ions could move faster, and the number of effective collisions might increase rapidly, resulting in partial coagulation of newly formed NPs with the larger size, causing a decrease in optical density. Therefore, the optimized temperature of 40 °C was chosen for the synthesis of CP-AgNPs.

#### 3.2.4. Effect of Reaction Time on the Synthesis of AgNPs

The reaction time plays an important role in the formation of MNPs; the longer the reaction time, the higher absorption maximum was observed. The *C. procera* flower aqueous extract that reacts with silver nitrate changes from colorless to dark brown after 4 h. The absorbance was measured throughout a range of incubation times. It has been postulated that silver ion to silver rapid conversion occurs after 4 h of incubation, showing a high SPR peak (Figure 2D). In case the reaction time is longer than 4 h, the decrease in SPR peak and the slight shift in maximum wavelength toward larger values indicated the formation of a large size of CP-AgNPs. Therefore, the overall results indicated that the optimum concentration of AgNO_3_ (5 mM) with the ratio of extract to silver nitrate of 1:3 at 40 °C in 4 h could be yielded as successful and higher content of AgNPs.

### 3.3. Structural and Morphological Characterization of CP-AgNPs

**Figure 3:**
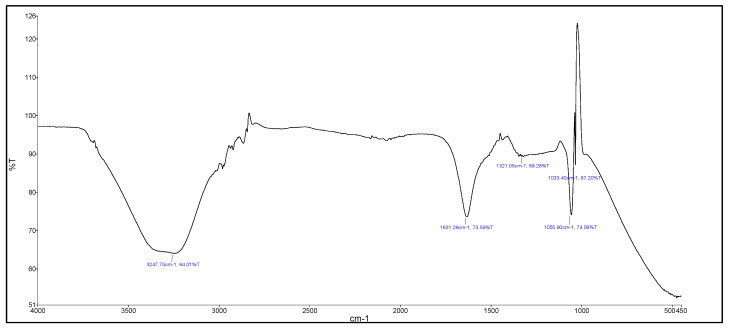
FTIR Analysis of synthesized AgNPs from *C. procera* flower extract.

Detailed characterizations of AgNPs were conducted in order to gain informed knowledge on the structural properties and morphology linked to CP-AgNPs. The FTIR spectra of the AgNPs synthesized employing *C. procera* flower aqueous extract are presented in Figure 3. The recorded spectra revealed demonstrated stretching vibrations bands representing the presence of the organic functional group. This includes 3247.75 cm^−1^, 1631.26 cm^−1^, 1321.05 cm^−1^, 1055.90 cm^−1^, and 1033.40 cm^−1^, ascribed to the vibrations of alcoholic O-H, C-N which displays protein, S=O stretching sulfone, C-N stretching amine, and the existence of flavanones adsorbed on NPs surface and C-O stretches, respectively. Moreover, absorption peaks at 1631 cm^−1^ and 1321 cm^−1^ represent the presence of NO_2_ of AgNO_3_. In addition, the strong interaction of water with silver could be the reason for OH stretching mode peaks at 2958 cm^−1^, 2921 cm^−1^, and 2852 cm^−1^ (minor peaks were not presented in the figure) [3]. However, several reports indicated that AgNPs are not expected to contribute to the spectral characterization. Therefore, these peaks might be originated from due to the capping and reducing agent CP, which have been shifted due to the formation of AgNPs. The overall FTIR spectra indicated capping of AgNPs by biomolecules present within CP flower extract. Furthermore, the average diameter, shape, and size morphology of CP-AgNPs were determined using TEM and DLS (Supplementary Figure S2A,B). TEM image at 50,000× illustrated the mono-dispersed similar size of the particles in the range of 23.30–90.86 nm. Whereas the DLS revealed median and mean particle diameter of 98.05 nm and 108.0 nm, respectively, with polydispersity of 0.239, which is much higher than the size observed using TEM, due to DLS measurement, it was carried out in an aqueous environment. As a result, there exists a tendency of measuring the hydration shell of water molecules surrounding the particles [6]. Moreover, the uniform dispersion with zeta potential of ~−35.1 mV resulted due to crystallite surface repulsive force from CP-capped AgNPs with stabilization by biomolecules.

**Figure 4:**
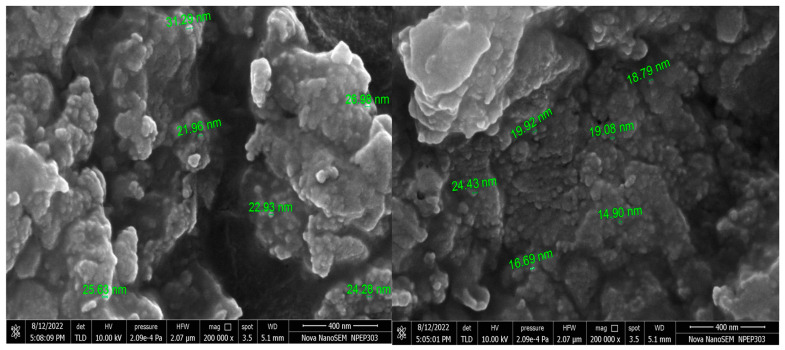
FESEM micrograph analysis of biosynthesized AgNPs using *C. procera* flower extract.

The CP-AgNPs surface morphology demonstrated their uniform spherical-to-cubic shape (Figure 4). The FE-SEM investigation demonstrated 14.90–31.29 nm of particle size. This result reaffirms that it may function as a reducing and capping agent in the synthesis of AgNPs. In similar investigation, surface morphology of *Pedalium murex* leaf extracts synthesized AgNPs is even spherical, with particle size ranging from 20 to 50 nm reported [33].

**Figure 5:**
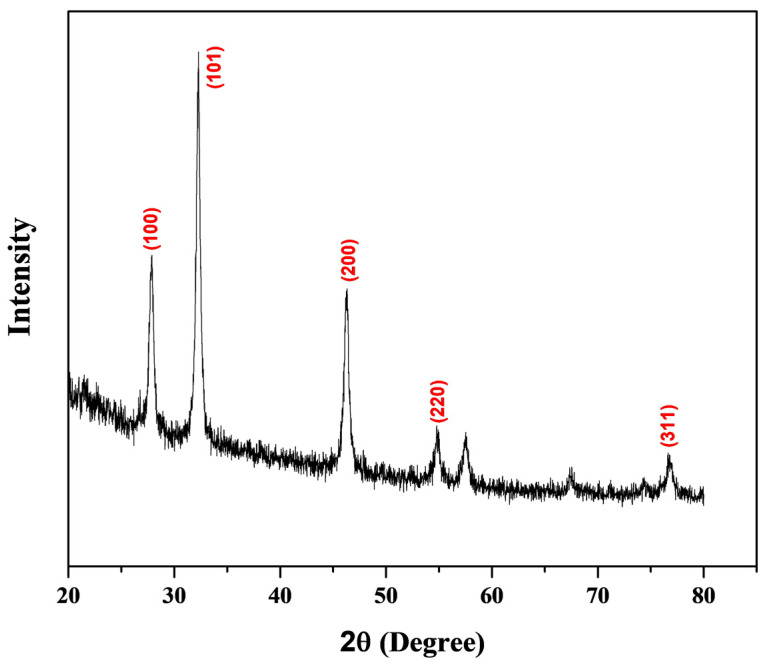
XRD pattern of green synthesized AgNPs using *C. procera* flower extract.

The X-ray powder diffraction patterns of biogenic-synthesized CP-AgNPs are presented in Figure 5. The positioning of the peaks CP-AgNPs is consistent with AgNPs. The varying angular positions and intensities of the diffracted peaks that appear from the projected monochromatic beam’s (θ) angles of incidence indicated reflection values of 38.18°, 44.37°, 66.46°, and 77.44°. These values are indexed in the planes of (100), (101), (2 0 0), (2 2 0), and (3 1 1). The results suggest that the resulting AgNPs are face-centered cubic (FCC) with a crystalline structure of 23.8 nm in size. Similar results were found after the synthesis of AgNPs *H. abyssinica* (Bruce) leaf [31] and *C. lancifolius* fruit extract [34] with an estimated average crystallographic size of 22.2 nm and 22.5 nm, respectively. Another recent study of AgNP syntheses resulted from the crystalline structure face-centered cubic to be 10 nm and 26.6 nm in size by employing *Fritillaria* flower extract [35] and leaf extract of *O. majorana,* respectively [36].

### 3.4. Antibacterial Activity of CP-AgNPs

**Figure 6:**
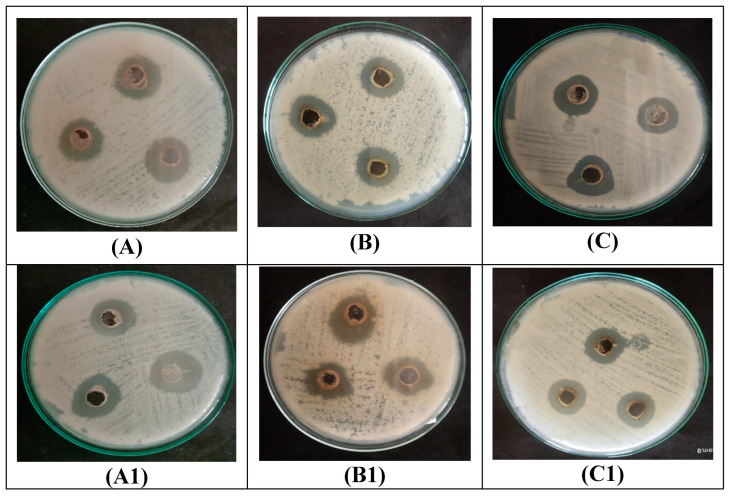

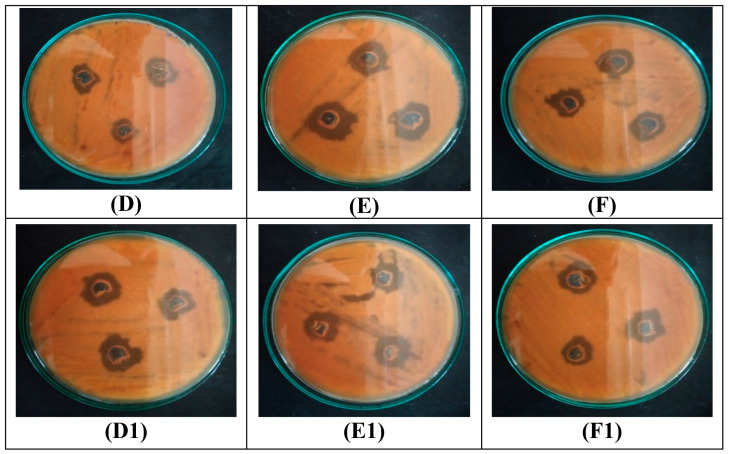
Antibacterial activity of synthesized AgNPs is presented while the illustration presents antibacterial activity of CP-AgNPs against *Bacillus subtilis* (**A**), *Bacillus megaterium* (**B**), and *Shigella flexneri* (**C**). Similarly, the antibiotic (Penicillin) was tested as a positive control against *Bacillus subtilis* (**A1**), *Bacillus megaterium* (**B1**), and *Shigella flexneri* (**C1**). Moreover, the antifungal activity of CP-AgNPs against *Aspergillus niger* (**D**), *penicillium crysogenum* (**E**), and *Trichoderma viride* (**F**), while antibiotic Fluconazole was tested as a positive control against *Aspergillus niger* (**D1**), *penicillium crysogenum* (**E1**), and *Trichoderma viride* (**F1**).

A zone of inhibition was observed in the area surrounding the test sample containing the well. The zone that has formed around the well indicates that the bacteria were susceptible to antibiotics (Figure 6). CP-AgNPs showed antibacterial activity against Gram-positive and Gram-negative bacteria. The Gram-negative bacteria strain (*Shigella flexneri*) represented a zone of 17 ± 0.2 mm, whereas Gram-positive bacteria (*Bacillus subtilis* and *Bacillus megaterium*) showed a zone of inhibition of 15.2 ± 0.4 and 14.7 ± 0.1 mm. These results indicate that the CP-AgNPs exhibit good antibacterial activity against a broad range of bacteria. The antibacterial activity of CP-AgNPs is due to their surface area that easily binds with microorganisms. The difference in the zone of inhibition is due to the different cell wall compositions of microorganisms. As Gram-negative bacteria consist of a single layer of peptidoglycan, whereas Gram-positive bacteria consist of a multilayer of peptidoglycan, Gram-positive bacteria consist of multilayers present on their cell wall. Due to the negative charge present in the cell wall, AgNPs can readily adhere to it by electrostatic attraction, changing the composition of the bacterial cell wall, which, in turn, affects its permeability. When the outcome is contrasted with earlier research on the latex of the *C. procera* AgNPs, the generated AgNPs are found to have antibacterial action against *E. coli*, *Pseudomonas aeuginosa*, *Serratia* sp. [37]. AgNPs made from newly harvested milky white latex of *C. gigantea* resulted in their ability against *Shigella* and *P. aeruginosa* [38]. Numerous studies have demonstrated that the shape, concentration, and type of AgNPs may be crucial in the control of bacteria. AgNPs are absorbed within the bacterial cells where they interact with the bacterial DNA and proteins that cause damage, which leads to increased oxidative stress through the production of reactive oxygen species. The reactive oxygen species production in bacterial mitochondria results in defective mitochondria, which prevents bacterial development [4].

### 3.5. Antifungal Activity of CP-AgNPs

The results showed that CP-AgNPs exhibit strong fungal growth inhibition against tested microorganisms, including *A. niger*, *P. crysogenum*, and *T. viride*. While antibiotic fluconazole was employed as the positive control, and demonstrated a zone of inhibition against *A. niger* around 18±0.1 mm, however, the zone for the *P. crysogenum* is 15.9 ± 0.4 mm, and 14.3 ± 0.2 mm against *T. viride* were significantly deferred, compared to the positive control (Figure 6). These results corroborated well with a previous study in which AgNPs synthesized using *C. procera* plant latex extract demonstrated antifungal activity against fungi *T. rubrum*, *C. albicans*, and *A. terreus* [37]. Additionally, the increase in reactive oxygen species leads to hydroxyl radical accumulation causing membrane damage, DNA fragmentation, and apoptosis of *Candida albicans* reported. Moreover, a previous study indicated lengthening of log phase in growth curve due to cell membrane disruption by AgNPs that leads to an impaired budding process in *Candida albicans*. Besides, free Ag^+^ ions can also attach to sulfur and phosphorus groups of cell membrane components and DNA leading to their degradation [1].

### 3.6. Antioxidant Activity of CP-AgNPs

Accumulation of unregulated hydrogen peroxide (H_2_O_2_) induces the formation of oxygen free radicals (peroxide and hydroxyl) that severely harms cell membranes in living systems. The capacity of CP-AgNPs to absorb H_2_O_2_ was measured spectrophotometrically. CP-AgNPs and ascorbic acid at 1 mg/mL demonstrated radical scavenging inhibition of 20.88% and 0.822%, respectively. The result indicated a remarkable reducing capacity of CP-AgNPs compared to the ascorbic acid due to quick dissolution of AgNPs that leads to higher cathepsin leakage from damaged lysosomes with the spontaneous clearance of turbid solution of H_2_O_2_ that was prepared in phosphate buffer. In this reaction, K^+^ and ion efflux may also play a role in the synthesis of superoxide and H_2_O_2_ in the mitochondrial membranes [39]. In a similar study, H_2_O_2_ scavenging activity of 93.32% for AgNPs biosynthesized using root extract of *Helicteres isora* [40]. In another study, green synthesized AgNPs indicated scavenging activity of 91.8% [6]. Human exposure to H_2_O_2_ is very high, because of its incorporation into personal care products as a bleaching agent or disinfectant. It is also used industrially for the bleaching of paper and pulp. This exposure promotes the generation of highly reactive hydroxyl radicals (OH) in the living system leading to cellular injury. In addition, H_2_O_2_ can also be generated in vivo due to the activities of some enzymes such as superoxide dismutase. It has the ability to cross the cell membrane, thereby causing the oxidation of several compounds in the cytosol. Moreover, the hydroxyl radicals and other reactive oxygen species have been implicated in several pathologies including intestinal diseases, Alzheimer’s disease, cancers, free radical-induced aging, inflammation, cell injury, and several other debilitating diseases. In view of these, it is necessary to limit exposure of living cells to H_2_O_2_, which can be achieved through the consumption of antioxidant-rich foods and appropriate treatment of environmental sources of H_2_O_2_, notably, drinking and wastewater [12]. The activities shown by CP-AgNPs in the present study indicate that NPs can serve as a potent nanotool to effectively scavenge H_2_O_2_ in wastewaters. In some other studies, NPs-based sensors have been developed for the detection and quantification of H_2_O_2_ [12].

### 3.7. Antidiabetic Activity of CP-AgNPs

The carbohydrate digestive enzymes, such as pancreatic α-amylase and intestinal α-glucosidase, are liable for the breakdown of oligosaccharides and disaccharides into monosaccharides suitable for absorption. The inhibition of these two digestive enzymes is especially useful for the treatment of non-insulin diabetes, because it will slow down the release of glucose in the blood. Moreover, the effective approach to lower blood glucose levels is to suppress the α-amylase enzyme. Alpha amylase inhibition decreases the rise in blood glucose levels by preventing the absorption of sugar from food. The results of α-amylase inhibitory activity of CP-AgNPs were higher (36.33%) than that of metformin (1.44%). Amylase is a crucial enzyme in the metabolism of carbohydrates. Starch blockers or amylase inhibitors inhibit the body from absorbing dietary starch. Therefore, consuming carbohydrates can reduce the rise in blood sugar levels [6]. In all the concentrations that were evaluated, the CP-AgNPs showed a higher level of α-amylase inhibitory activity than acarbose. The percentage of inhibition increased in a dose-dependent manner with increasing silver nanoparticle concentration. Previous investigations showed an inhibitory action of AgNPs synthesized using *O. basilicum* and *O. sanctum* leaf extract compared with α-amylase enzymes at 89.31 5.32% and 79.74 9.51%, respectively [41].

### 3.8. Anti-Inflammatory Activity of Synthesized AgNPs

#### 3.8.1. Inhibition of Albumin Denaturation

Albumin denaturation inhibitory activity of CP-AgNPs is the reaction process that mimics living tissues to stimuli evoked by inflammatory agents such as physical injuries, heat microbial infections, and noxious chemical irritation. The response of cells toward inflammation leads to certain pathophysiological functions. Inflammation has been associated with pathogenesis of several diseases such as arthritics, stroke, and cancer. In comparison to standard drug aspirin, the synthesized CP-AgNPs prevented albumin denaturation by 68.63%. The increases in absorbance of test samples, compared to controls, showed that CP-AgNPs and the standard drug inhibited heat-induced protein denaturation and stabilized the protein. Moreover, the results revealed that the CP-AgNPs efficiently reduced the induced albumin denaturation. The CP-AgNPs significantly reduced the heat-induced hemolysis and stabilized the RBC membrane. In a similar study, green synthesized AgNPs, and the drug demonstrated concentration-dependent protein [42]. The results were in contrast to a study on the green synthesis of AgNPs that inhibit albumin denaturation, which showed that an increase in AgNPs concentration lowers the activity and results in IC50 values of 65.2 and 53.8 g/mL, respectively [43].

#### 3.8.2. Membrane Stabilization Test

The hypotonic solution has a hemolytic effect. Hemolysis occurs when there is accumulation of excessive fluid into the cells resulting in rupture of RBCs membrane, and when the RBCs membrane gets injured, it will make the cell more suspectable to secondary damage. This damage occurs by free radical-induced lipid peroxidation. Moreover, the escape of serum protein and fluids from the tissue can be prevented by membrane stabilization. Therefore, the effect of CP-AgNPs on membrane stabilization was studied using fresh human blood. The suppression of hypotonicity-induced RBCs membrane lysis and membrane stabilization using CP-AgNPs, and aspirin, were determined to quantify the anti-inflammatory activity. A 1 µg/mL sample of synthesized CP-AgNPs and standard exhibited 20% and 33% of membrane stabilization, respectively. The investigated sample, concentration gradient, and environmental factors all affect how much membrane lysis is suppressing. The difference between aspirin’s result of 85.89% and that of earlier studies’ 84.18% aspirin and biosynthesized AgNPs utilizing *Calophyllum tomentosum* leaves extract could be the consequence of different experimental circumstances [44]. However, results were contrasted with a previous report indicating that the chitosan-derived AgNPs showed a 62.74% membrane stabilization activity [45]. Several studies tested the biocompatibility of MNPs against mammalian and cancer cells. Interestingly, AgNPs demonstrated dose-dependent cytotoxicity against mammalian cells with significant anticancer activity indicating that they can be use as biomedicine in the near future [5,6]. Though recently, topical application on AgNPs in semisolid gel form or in incorporated within composite showed efficacy over tested animals; however, human clinical investigation is necessary for approval from regulatory agencies of various countries [1].

## 4. Conclusions

The production of nanoparticles using biological agents is environmentally friendly, inexpensive, and feasible at room temperature. In the current investigation, phytochemicals found in *Calotropis procera* flower extracts function as both reducing and stabilizing agents. In conclusion, we have successfully synthesized AgNPs. The CP-AgNPs exhibited improved biomimetic attributes that encourage further animal and clinical investigation.

## Data Availability

Not applicable.

## Supplementary Materials

The following supporting information can be downloaded at: https://www.mdpi.com/article/10.3390/ma16114058/s1.

## References

[1] Singh S., Nwabor O.F., Sukri D.M., Wunnoo S., Dumjun K., Lethongkam S., Kusolphat P., Hemtanon N., Klinprathum K., Sunghan J. (2022). Poly (vinyl alcohol) copolymerized with xanthan gum/hypromellose/sodium carboxymethyl cellulose dermal dressings functionalized with biogenic nanostructured materials for antibacterial and wound healing application. Int. J. Biol. Macromol..

[2] Suriyakala G., Sathiyaraj S., Devanesan S., AlSalhi M.S., Rajasekar A., Maruthamuthu M.K., Babujanarthanam R. (2022). Phytosynthesis of silver nanoparticles from Jatropha integerrima Jacq. flower extract and their possible applications as antibacterial and antioxidant agent. Saudi J. Biol. Sci..

[3] Ontong J.C., Singh S., Nwabor O.F., Chusri S., Voravuthikunchai S.P. (2020). Potential of antimicrobial topical gel with synthesized biogenic silver nanoparticle using Rhodomyrtus tomentosa leaf extract and silk sericin. Biotechnol. Lett..

[4] Singh S., Chunglok W., Nwabor O.F., Ushir Y.V., Singh S., Panpipat W. (2022). Hydrophilic Biopolymer Matrix Antibacterial Peel-off Facial Mask Functionalized with Biogenic Nanostructured Material for Cosmeceutical Applications. J. Polym. Environ..

[5] Jayeoye T.J., Eze F.N., Singh S., Olatunde O.O., Benjakul S., Rujiralai T. (2021). Synthesis of gold nanoparticles/polyaniline boronic acid/sodium alginate aqueous nanocomposite based on chemical oxidative polymerization for biological applications. Int. J. Biol. Macromol..

[6] Jayeoye T.J., Eze F.N., Olatunde O.O., Singh S., Zuo J., Olatunji O.J. (2021). Multifarious Biological Applications and Toxic Hg(2+) Sensing Potentiality of Biogenic Silver Nanoparticles Based on Securidaca inappendiculata Hassk Stem Extract. Int. J. Nanomed..

[7] Nwabor O.F., Singh S., Wunnoo S., Lerwittayanon K., Voravuthikunchai S.P. (2021). Facile deposition of biogenic silver nanoparticles on porous alumina discs, an efficient antimicrobial, antibiofilm, and antifouling strategy for functional contact surfaces. Biofouling.

[8] Nwabor O.F., Singh S., Ontong J.C., Vongkamjan K., Voravuthikunchai S.P. (2021). Valorization of Wastepaper Through Antimicrobial Functionalization with Biogenic Silver Nanoparticles, a Sustainable Packaging Composite. Waste Biomass Valorization.

[9] Nwabor O.F., Singh S., Paosen S., Vongkamjan K., Voravuthikunchai S.P. (2020). Enhancement of food shelf life with polyvinyl alcohol-chitosan nanocomposite films from bioactive Eucalyptus leaf extracts. Food Biosci..

[10] Syukri D.M., Nwabor O.F., Singh S., Ontong J.C., Wunnoo S., Paosen S., Munah S., Voravuthikunchai S.P. (2020). Antibacterial-coated silk surgical sutures by ex situ deposition of silver nanoparticles synthesized with Eucalyptus camaldulensis eradicates infections. J. Microbiol. Methods.

[11] Syukri D.M., Nwabor O.F., Singh S., Voravuthikunchai S.P. (2021). Antibacterial functionalization of nylon monofilament surgical sutures through in situ deposition of biogenic silver nanoparticles. Surf. Coat. Technol..

[12] Kumar A., Shah S.R., Jayeoye T.J., Kumar A., Parihar A., Prajapati B.G., Singh S., Kapoor D. (2023). Biogenic metallic nanoparticles: Biomedical, analytical, food preservation, and applications in other consumable products. Front. Nanotechnol..

[13] Bhat M., Chakraborty B., Kumar R.S., Almansour A.I., Arumugam N., Kotresha D., Pallavi S., Dhanyakumara S., Shashiraj K., Nayaka S. (2021). Biogenic synthesis, characterization and antimicrobial activity of Ixora brachypoda (DC) leaf extract mediated silver nanoparticles. J. King Saud Univ.-Sci..

[14] Iravani S., Korbekandi H., Mirmohammadi S.V., Zolfaghari B. (2014). Synthesis of silver nanoparticles: Chemical, physical and biological methods. Res. Pharm. Sci..

[15] Zhang X.-F., Liu Z.-G., Shen W., Gurunathan S. (2016). Silver nanoparticles: Synthesis, characterization, properties, applications, and therapeutic approaches. Int. J. Mol. Sci..

[16] El-Khatib A.M., Badawi M.S., Ghatass Z., Mohamed M., Elkhatib M. (2018). Synthesize of silver nanoparticles by arc discharge method using two different rotational electrode shapes. J. Clust. Sci..

[17] Weerasinghe J., Li W., Zhou R., Zhou R., Gissibl A., Sonar P., Speight R., Vasilev K., Ostrikov K. (2020). Bactericidal silver nanoparticles by atmospheric pressure solution plasma processing. Nanomaterials.

[18] Gudikandula K., Charya Maringanti S. (2016). Synthesis of silver nanoparticles by chemical and biological methods and their antimicrobial properties. J. Exp. Nanosci..

[19] Chandraker S.K., Ghosh M.K., Lal M., Shukla R. (2021). A review on plant-mediated synthesis of silver nanoparticles, their characterization and applications. Nano Express.

[20] Bordiwala R.V. (2023). Green Synthesis and Applications of Metal Nanoparticles—A Review article. Results Chem..

[21] Al-Rowaily S.L., Abd-ElGawad A.M., Assaeed A.M., Elgamal A.M., Gendy A.E.-N.G.E., Mohamed T.A., Dar B.A., Mohamed T.K., Elshamy A.I. (2020). Essential oil of Calotropis procera: Comparative chemical profiles, antimicrobial activity, and allelopathic potential on weeds. Molecules.

[22] Chidrawar V.R., Singh S., Jayeoye T.J., Dodiya R., Samee W., Chittasupho C. (2023). Porous Swellable Hypromellose Composite Fortified with Eucalyptus camaldulensis Leaf Hydrophobic/Hydrophilic Phenolic-rich Extract to Mitigate Dermal Wound Infections. J. Polym. Environ..

[23] Singh S., Chidrawar V.R., Hermawan D., Dodiya R., Samee W., Ontong J.C., Ushir Y.V., Prajapati B.G., Chittasupho C. (2023). Hypromellose Highly Swellable Composite Fortified with Psidium Guajava Leaf Phenolic-rich Extract for Antioxidative, Antibacterial, Anti-inflammatory, Anti-melanogenesis, and Hemostasis Applications. J. Polym. Environ..

[24] Paramasivam D., Balasubramanian B., Suresh R., Kumaravelu J., Vellingiri M.M., Liu W.-C., Meyyazhagan A., Alanazi A.M., Rengasamy K.R., Arumugam V.A. (2023). One-Pot Synthesis of Silver Nanoparticles Derived from Aqueous Leaf Extract of Ageratum conyzoides and Their Biological Efficacy. Antibiotics.

[25] Ajaykumar A.P., Mathew A., Chandni A.P., Varma S.R., Jayaraj K.N., Sabira O., Rasheed V.A., Binitha V.S., Swaminathan T.R., Basheer V.S. (2023). Green Synthesis of Silver Nanoparticles Using the Leaf Extract of the Medicinal Plant, Uvaria narum and Its Antibacterial, Antiangiogenic, Anticancer and Catalytic Properties. Antibiotics.

[26] Alowaiesh B.F., Alhaithloul H.A.S., Saad A.M., Hassanin A.A. (2023). Green Biogenic of Silver Nanoparticles Using Polyphenolic Extract of Olive Leaf Wastes with Focus on Their Anticancer and Antimicrobial Activities. Plants.

[27] Meena A., Yadav A., Meda M. (2011). Ayurvedic uses and pharmacological activities of *Calotropis procera* Linn. Asian J. Tradit. Med..

[28] Morsy N., Sherif E., Abdel-rassol T. (2016). Phytochemical analysis of *Calotropis procera* with antimicrobial activity investigation. Main Group Chem..

[29] Alzubaidi A.K., Al-Kaabi W.J., Ali A.A., Albukhaty S., Al-Karagoly H., Sulaiman G.M., Asiri M., Khane Y. (2023). Green Synthesis and Characterization of Silver Nanoparticles Using Flaxseed Extract and Evaluation of Their Antibacterial and Antioxidant Activities. Appl. Sci..

[30] Mukaratirwa-Muchanyereyi N., Gusha C., Mujuru M., Guyo U., Nyoni S. (2022). Synthesis of silver nanoparticles using plant extracts from *Erythrina abyssinica* aerial parts and assessment of their anti-bacterial and anti-oxidant activities. Results Chem..

[31] Melkamu W.W., Bitew L.T. (2021). Green synthesis of silver nanoparticles using *Hagenia abyssinica* (Bruce) JF Gmel plant leaf extract and their antibacterial and anti-oxidant activities. Heliyon.

[32] Patil L., Shet A., Tennalli G., Achappa S., Hombalimath V., Deshannavar U. (2020). Optimization of Process Parameters for Synthesis of Silver Nanoparticles Using Leaf Extract of Tridax Procumbent and Its Biotechnological Applications. Int. J. Sci. Technol. Res..

[33] Anandalakshmi K., Venugobal J., Ramasamy V. (2016). Characterization of silver nanoparticles by green synthesis method using *Pedalium murex* leaf extract and their antibacterial activity. Appl. Nanosci..

[34] Oves M., Rauf M.A., Aslam M., Qari H.A., Sonbol H., Ahmad I., Zaman G.S., Saeed M. (2022). Green synthesis of silver nanoparticles by *Conocarpus lancifolius* plant extract and their antimicrobial and anticancer activities. Saudi J. Biol. Sci..

[35] Hemmati S., Rashtiani A., Zangeneh M.M., Mohammadi P., Zangeneh A., Veisi H. (2019). Green synthesis and characterization of silver nanoparticles using *Fritillaria* flower extract and their antibacterial activity against some human pathogens. Polyhedron.

[36] Yassin M.T., Mostafa A.A.-F., Al-Askar A.A., Al-Otibi F.O. (2022). Facile green synthesis of silver nanoparticles using aqueous leaf extract of *Origanum majorana* with potential bioactivity against multidrug resistant bacterial strains. Crystals.

[37] Mohamed N.H., Ismail M.A., Abdel-Mageed W.M., Shoreit A.A.M. (2014). Antimicrobial activity of latex silver nanoparticles using Calotropis procera. Asian Pac. J. Trop. Biomed..

[38] Rajkuberan C., Sudha K., Sathishkumar G., Sivaramakrishnan S. (2015). Antibacterial and cytotoxic potential of silver nanoparticles synthesized using latex of *Calotropis gigantea* L.. Spectrochim. Acta Part A Mol. Biomol. Spectrosc..

[39] He W., Zhou Y.-T., Wamer W.G., Boudreau M.D., Yin J.-J. (2012). Mechanisms of the pH dependent generation of hydroxyl radicals and oxygen induced by Ag nanoparticles. Biomaterials.

[40] Bhakya S., Muthukrishnan S., Sukumaran M., Muthukumar M. (2016). Biogenic synthesis of silver nanoparticles and their antioxidant and antibacterial activity. Appl. Nanosci..

[41] Malapermal V., Botha I., Krishna S.B.N., Mbatha J.N. (2017). Enhancing antidiabetic and antimicrobial performance of Ocimum basilicum, and *Ocimum sanctum* (L.) using silver nanoparticles. Saudi J. Biol. Sci..

[42] Prabakaran A., Mani N. (2019). Anti-inflammatory activity of silver nanoparticles synthesized from Eichhornia crassipes: An in vitro study. J. Pharmacogn. Phytochem..

[43] Kedi P.B.E., Meva F.E., Kotsedi L., Nguemfo E.L., Zangueu C.B., Ntoumba A.A., Mohamed H.E.A., Dongmo A.B., Maaza M. (2018). Eco-friendly synthesis, characterization, in vitro and in vivo anti-inflammatory activity of silver nanoparticle-mediated Selaginella myosurus aqueous extract. Int. J. Nanomed..

[44] Govindappa M., Hemashekhar B., Arthikala M.-K., Rai V.R., Ramachandra Y. (2018). Characterization, antibacterial, antioxidant, antidiabetic, anti-inflammatory and antityrosinase activity of green synthesized silver nanoparticles using *Calophyllum tomentosum* leaves extract. Results Phys..

[45] Hebeish A., Ramadan M., Montaser A., Farag A.M. (2014). Preparation, characterization and antibacterial activity of chitosan-g-poly acrylonitrile/silver nanocomposite. Int. J. Biol. Macromol..

