# Supplementary material for: Biogenic Fabrication of Silver Nanoparticles Using Calotropis procera Flower Extract with Enhanced Biomimetics Attributes
Source: Materials (Basel). 2023 May 30;16(11):4058. doi: 10.3390/ma16114058 (PMC10254777; doi:10.3390/ma16114058)
Supplement: Supplementary file 1 [file materials-16-04058-s001.zip › materials-2388456-supplementary.pdf]

Biogenic Fabrication of Silver Nanoparticles Using *Calotropis procera* Flower Extract with Enhanced Biomimetics Attributes

Pooja V Nagime[1*], Sudarshan Singh[2], Nishat M Shaikh[1], Komal S Gomare[1], Havagiray Chitme[3*], Basel A. Abdel-Wahab[4], Yahya S. Alqahtany[5] Masood Medleri Khateeb[4], Mohammed Shafiuddin Habeeb[4], and Marwa B. Bakir[6]

[1] Department of Biotechnology, Dayanand Science College, Latur, Maharashtra, 413512, India [PVN: poojanagime1010@gmail.com; NMSN: nishatshaikh2017@gmail.com; KSG: komalgomare2007@gmail.com]

[2] Department of Pharmaceutical Sciences, Faculty of Pharmacy, Chiang Mai University, Chiang Mai 50200, Thailand [SS: sudarshansingh83@hotmail.com]

[3] Faculty of Pharmacy, DIT University, Dehradun 248009, Uttarakhand, India [HC: hrchitme@gmail.com]

[4] Department of Pharmacology, College of Pharmacy, Najran University, Najran, Saudi Arabia (BAW: babdelnaem@nu.edu.sa; MMK: mmkhateeb@nu.edu.sa; MSH: mshabeeb@nu.edu.sa]

[5] Department of Pharmaceutical Chemistry, College of Pharmacy, Najran University, Najran, Saudi Arabia [YSA: ysalqahtany@nu.edu.sa]

[6] Department of Pharmacology, College of Medicine, Najran University, Najran, Saudi Arabia; [MBB: mbbakir@nu.edu.sa]

*Correspondence: poojanagime1010@gmail.com; hrchitme@gmail.com

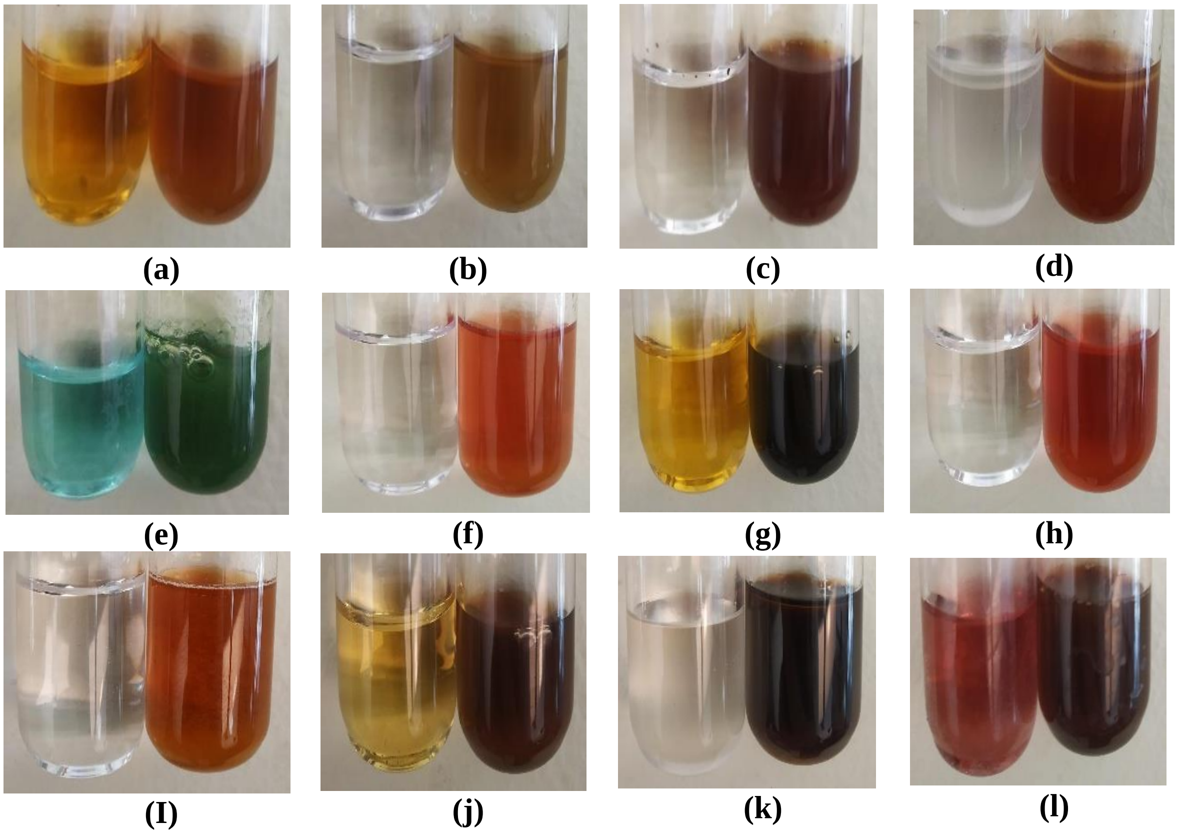

Supplementary Figure S1. Phytochemical analysis of *Calotropis procera* flower extract representing traces of alkaloid (a), anthocyanin (b), glycoside (c), carbohydrate (d), anthoquinone (e), diterpene (f), flavonoid (g), phenol (h), phalobatanin (i), protein (j), tannin (k), terpenoid test (l).

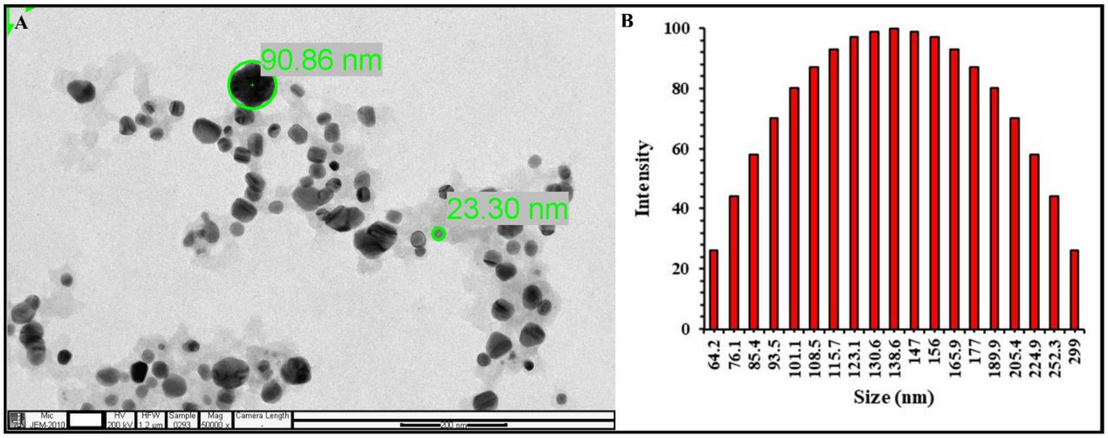

Supplementary Figure S2. TEM images showing the spherical to cubic shaped CP-AgNPs at 50000 X (A). Particle size histogram of silver nanoparticles using DLS (B).

Supplementary Table S1 Results of phytochemical analysis of *Calotropis procera* flower extract

Sr No.	Phytocompound	Reagent	Result
1	Alkaloid	Wanger's reagent	+
2	Anthocyanin	NaOH reagent	+
3	Anthraquinone	Borntrager's reagent	+
4	Carbohydrates	Molish test	-
5	Diterpene	Copper acetate	+
6	Flavonoid	Alkaline reagent	-
7	Glycoside	Sulphuric acid reagent	+
8	Phenol	Ferric chloride reagent	+
9	Phlobatanin	HCL reagent	+
10	Protein	Xanthproteic reagent	+
11	Tannin	Braymer's reagent	-
12	Terpenoid	Salkowaski reagent	+

Note: Present (+); Absent (-).
